# Antidiabetic properties of dietary flavonoids: a cellular mechanism review

**DOI:** 10.1186/s12986-015-0057-7

**Published:** 2015-12-23

**Authors:** Ramachandran Vinayagam, Baojun Xu

**Affiliations:** Food Science and Technology Program, Beijing Normal University–Hong Kong Baptist University United International College, 28, Jinfeng Road, Tangjiawan, Zhuhai, Guangdong 519085 China

**Keywords:** Dietary flavonoids, Diabetes mellitus, Glycemic control, Insulin secretion, Hyperglycemia

## Abstract

**Background:**

Natural food products have been used for combating human diseases for thousands of years. Naturally occurring flavonoids including flavones, flavonols, flavanones, flavonols, isoflavones and anthocyanidins have been proposed as effective supplements for management and prevention of diabetes and its long-term complications based on *in vitro* and animal models.

**Aim:**

To summarize the roles of dietary flavonoids in diabetes management and their molecular mechanisms.

**Findings:**

Tremendous studies have found that flavonoids originated from foods could improve glucose metabolism, lipid profile, regulating the hormones and enzymes in human body, further protecting human being from diseases like obesity, diabetes and their complications.

**Conclusion:**

In the current review, we summarize recent progress in understanding the biological action, mechanism and therapeutic potential of the dietary flavonoids and its subsequent clinical outcomes in the field of drug discovery in management of diabetes mellitus.

## Background

Type 2 diabetes mellitus (DM) is characterized by impaired insulin secretion, and increased insulin resistance (or resistance to insulin mediated glucose disposal). DM is possibly one of the world’s fast growing diseases [1] and disabling micro and macrovascular complications [2]. It has been estimated that nearly 592 million adults become diabetic patients by the year 2035 due to aging, high population growth size, increased urbanization, high prevalence of obesity, rise in living standards and the spread of calorie rich, fatty and fast foods [3]. Although many drugs are commercially available for treating the diseases, many of them are out of reach for a significant proportion of the population and are beset with some adverse effects [4]. The use of medicinal plants and their phytochemicals for treating diabetes is not just a search for safer alternatives to pharmaceuticals, which transiently lower the blood glucose, preventing heart disease and high blood pressure, and also enhancing the antioxidant system, insulin action and secretion [5]. Phyto-constituents have always guided the search for clinical trials, drug discovery and development. Hence, there is search for dietary constituents as well as natural antioxidants that can regulate blood glucose levels.

In the present review the connection between flavonoids and DM is focused on the basis of recent studies. The anti-diabetic activities of the flavonoids found in dietary plants, fruits, and summarize the underlying molecular signaling of the dietary flavonoids using *in vitro* and *in vivo* models to clarify their anti-diabetic effects.

## Molecular mechanisms of insulin resistance

The molecular levels, the mechanisms underlying insulin resistance are being explored. Several mechanisms including abnormal insulin production, mutations in insulin receptor (IR) and its substrates, and insulin antagonists have been proposed, but it is now clearly known that defects in post-receptor signaling are the major cause of insulin resistance in target tissues [6]. Reduced expression, diminished tyrosine phosphorylation, or increased degradation of early insulin signaling molecules have been implicated.

Serine/threonine phosphorylation of IRS proteins can inhibit their activity and block downstream signaling. Various kinases including stress - activated protein kinase, c-Jun N-terminal kinase (JNK), inhibitor of nuclear factor kappa B (NF-κB) kinase (IκB), and protein kinase C (PKC) can phosphorylate IRS-1 and 2 at specific serine and threonine residues, leading to inhibition of insulin signaling [7]. Another underlying mechanism is the induction of inhibitory factors such as suppressors of cytokine signaling (SOCS-1 and 3). SOCS proteins block insulin signaling via competition with IRS-1 for association with the IR and by augmentation of proteosomal degradation of IRS-1 [8].

Increased activity of phosphatases which dephosphorylate intermediate signaling molecules can also inhibit the insulin signal pathway [9]. Several phosphatases have been implicated as inhibitors of insulin action. Protein tyrosine phosphatases (eg PTP1B) have negative effects on insulin signaling and are suggested to be major regulators of insulin signaling [10]. Other phosphatases such as phosphatase and tensin homologue (PTEN) which inactivates PI3-K and SH2-containing inositol 5′ phosphatase-2 [SHIP 2] have been shown to have a negative role on insulin signaling [11, 12].

## Regulation of lipogenesis in adipocytes

Adipocyte transcription factors such as peroxisome proliferator-activated receptor gamma (PPARγ), sterol regulatory element-binding protein (SREBP-1c), adipokines like resistin, play crucial roles in adipocyte differentiation, adipogenesis, and accumulation of cellular lipid droplets.

PPARγ, a nuclear hormone receptor, is mainly expressed in adipose tissue and to a lesser extent in colon, immune cells and retina [13]. It plays a critical role in peripheral glucose homeostasis and energy metabolism and it also has been implicated in modulating adipogenesis and insulin sensitivity in vivo. PPAR*γ*, induces glucose and fatty acid uptake by directly or indirectly enhancing the transcription of genes encoding proteins such as, fatty acid binding proteins (aP2), GLUT4 [14], fatty acid transport proteins, and acyl-CoA synthetase. Recent studies indicate that partial PPAR*γ* antagonism by various flavonoids may be beneficial in improving insulin sensitivity and may also inhibit adipocyte differentiation and lipid accumulation [15].

Adiponectin is supposed to improve primarily glucose and lipid metabolism. Adiponectin also inhibits the expression of several pro-inflammatory cytokines [16], including tumor necrosis factor. Among other factors released within adipose tissue, tumor necrosis factor alpha (TNF*α*) promotes lipolysis and increases (free fatty acids) FFAs; both TNF*α* and interleukin 6 (IL-6) are related to mitochondrial dysfunction. Increased expression of TNF*α* and relatively low levels of adiponectin promote lipolysis and FFA release.

Leptin is an adipocyte-secreted hormone whose absence leads to dramatic metabolic derangements. Leptin regulates food intake at the level of hypothalamus and stimulates FFA oxidation in peripheral tissues to avoid lipid deposition. Insulin resistance in animal results in hyperleptinemia induces leptin resistance and causes lipotoxicity [17]. Leptin resistance causes a rise in FFA release to circulation followed by triglycerides (TG) synthesis (fatty liver) and decreased FFA oxidation in liver [18].

## Flavonoids and their effects on diabetes

Flavonoids represent a large class of at least 6000 phenolic compounds found in fruits, vegetables, nuts, grain seeds, cocoa, chocolate, tea, soy, red wine, herbs and beverage products. Structurally, flavonoids consist of two aromatic rings (A and B rings) linked by a 3-carbon chain that forms an oxygenated heterocyclic ring (C ring). There are six subclasses of flavonoids including flavones, flavonols, flavanones, flavonols, isoflavones and anthocyanidins based on differences in generic structure of the C ring, functional groups on the rings and the position at which the B ring is attached to the C ring. Within each subclass, individual compounds are characterized by specific hydroxylation and conjugation patterns. Flavonoids that have the ability to scavenge free radicals and chelate metals [19]. Given the hypothesized relation between diabetes and inflammation [20, 21] and the potential for flavonoids to protect the body against free radicals and other pro-oxidative compounds [22, 23], it is biologically plausible that consumption of flavonoids or flavonoid-rich foods may reduce the risk of diabetes [24, 25]. New concepts have appeared with this trend, such as nutraceuticals, nutritional therapy, phytonutrients and phytotherapy. This functional foods and phytomedicines play positive roles in maintaining blood glucose levels, glucose uptake and insulin secretion and modulating immune function to prevent specific DM [26, 27]. In current years various approaches have been made to utilize the flavonoids in vitro and in vivo models by incorporating few novel methods to improve its antidiabetic activity. They are categorized in Table 1.

**Table 1:** Important anti-diabetic potential and the underlying mechanism of dietary flavonoids

Structure of flavonoid (s)	Plants/Dietary source	Specific mechanism of action	Model	References
Diosmin	*Scrophularia nodosa* L., and citrus fruits	Stimulating the insulin production from the existing β-cells of pancreas.	STZ-nicotinamide-induced diabetic rats.	Srinivasan and Pari 2010, 2012, 2013 [30, 31, 33]
		↓ Lipids profile, improving lipid metabolizing enzymes, antioxidant and ↓ lipid peroxidation.		
		↓ Blood glucose, lipid peroxides, NO and ↑ body weight.	STZ-induced diabetic neuropathy rats	Jain 2014 [32]
		↓ Glycation.	STZ-diabetic rats	Vertommen 1994 [34]
Fisetin	Strawberries, onion and persimmon	Improved glucose homeostasis.	STZ-diabetic rats	Prasath 2014 [38]
		↑ Glycolysis, ↓ gluconeogenesis.	STZ-diabetic rats	Prasath and Subramanian 2011, 2013 [39, 45]
		↓ Blood glucose, HbA1c, NF-κB p65, IL-1β and nitric oxide.		
		Suppress HATs, particularly p300, leading to deacetylation of the p65 subunit of NF-κB.	THP-1 cell line	Kim 2012 [40]
		Reduced cataract formation.	STZ-diabetic cataract in mice	Kan 2015 [41]
		↑ HDL, ↓ LDL and VLDL.	STZ-diabetic rats	Prasath and Subramanian 2014 [42]
		Stimulate the NF-κB pathway, downregulated of adhesion molecules, inhibition of monocyte-endothelial adhesion.	Human umbilical vein endothelial cells and C57BL/6 mice	Kwak 2014 [43]
		Improved glycemic and antioxidant status.	STZ-diabetic rats.	Prasath and Subramanian 2013 [44]
		↑ Mitochondrial function.		
		↓ Level methylglyoxal-dependent protein glycation.	C57BL/6-Ins2 Akita mice	Maher 2011 [46]
Morin	*Prunus dulcis* (Mill.) D.A. Webb.*, Chlorophora tinctoria* (L.) Gaud.*, Psidium guajava* L.*,* fruits and wine	↑ Insulin sensitivity and ↓ oxidative stress.	HFD-STZ-induced diabetic rats	Sendrayaperumal 2014 [49]
		Inhibition of PTP1B, which behaves as an activator and sensitizer of the insulin receptor stimulating the metabolic pathways.	HepG2 cell line	Paoli 2013 [52]
		Preventing the destruction of β-cells of the islets of Langerhans.	STZ induced diabetic rats	Vanitha 2014 [53]
		Inhibition in ROS generation, translocation of apoptotic proteins, up-regulation of antioxidantgenes and Bcl-2 gene expression.	Hepatocytes cell line	Kapoor 2012 [54]
Eriodictyol	*Eriodictyon californicum* (Hook. & Arn.) Torr*, Millettia duchesnei* De Wild., *Eupatorium arnottianum* Griseb and lemon	↑ Glucose uptake and improve insulin resistance	HepG2 cell line	Zhang 2012 [56]
		↓ TNFα, ICAM-1, VEGF, and eNOS.	STZ-induced diabetic rats	Bucolo 2012 [57]
Hesperidin	Orange citrus aurantium	Down-regulates generation of free radical, release of cytokines (TNF- α and IL-1β).	Neuropathy rats	Visnagri 2014 [60]
		Anti-angiogenic, anti-inflammatory effects.	STZ-induced diabetic rats.	Shi 2012 [62]
		↓ Blood glucose by altering the activity of glucose-regulating enzymes.	STZ-induced type 1 diabetic rats	Akiyama 2010 [63]
		Reduced oxidative stress, apoptosis and improving cardiac function via the PPAR-γ pathway.	STZ-isoproternol induced diabetic rats	Yo 2014 [64]
		↓ Inflammatory cytokies	HFD-STZ-induced type 2 diabetic rats.	Mahmoud 2012 [65]
		↓ HbA1c, glucose, CES LDL, TC, TG levels, systolic and diastolic blood pressure.	STZ-nicotinamide induced myocardial infarction in diabetes in rats	Kakadiya 2010 [66]
		Regulation of glucose and lipid metabolism.	Goto-Kakizaki type 2 diabetes rats	Akiyama 2009 [67]
		Regulation of glycolysis, gluconeogenesis, hepatic glycogen stores.	C57BL/KsJ-db/db mice	Jung 2014 [68]
		↓ Lipid peroxidation, ↑ GSH, GR and GST	STZ-induced diabetes rats	Ashafaq 2014 [69]
Naringenin	*Cochlospermum vitifolium* (Willd.) Spreng.*,* grapefruits, oranges and tomatoes	Inhibition of intestinal α-glucosidase activity.	High HFD-STZ induced diabetic rats.	Priscilla 2014 [73]
		Reduced oxidative damage	STZ-induced diabetes rats	Fallahi 2012 [75]
		↓ Cholesterol and cholesterol ester synthesis.	High-fat induced diabetic mice	Mulvihill 2009 [80]
		Improved overall insulin sensitivity and glucose tolerance.		
		Suppressed monocyte chemoattractant protein-1 and inhibition of c-Jun NH2-terminal kinase pathway.	HFD-induced obesity	Yoshida 2014 [82]
		↓ Hyperglycemia and ↑ antioxidant enzyme (SOD).	STZ-induced hyperalgesia and allodynia in rats	Hasanein 2014 [83]
		Stimulated insulin secretion	INS-1E cells	Bhattacharya 2014 [84]
		Decreased fasting glucose and inflammatory cytokines.	HFD-fed mice	Yoshida 2013 [85]
		↓ Oxidative stress.	STZ-induced diabetes rats	Rahigude 2012 [86]
Apigenin	*Hypericum perforatum* L., *Matricaria chamomilla L.,* parsley, onions, oranges, tea, chamomile and wheat sprouts.	↓ Glucose and G-6-Pase activity and ↑ antioxidant enzymes.	Alloxan-induced diabetic mice	Panda and Kar 2007 [88]
		↓ Apoptosis, ↑ antioxidant and mitochondrial protection.	HIT-T15 pancreatic β-cells	Suh 2012 [90]
		Inhibition TNF-α and IL-1β-induced activation of NF-κB.	Human THP-1 monotypic cells	Zhang 2014 [92]
		Inhibition the expression of VCAM1, IKKα and IKKepsilon/IKKi.	Human endothelial cells	Yamagata 2010 [93]
		Insulin-secretagogue.	Male Wistar rats	Cazarolli 2009 [94]
Baicalein	*Scutellaria baicalensis* Georgi and *Scutellaria lateriflora* L.	Improved glucose tolerance, and islet β-cell survival and mass.	HFD-induced obese mice.	Fu 2014 [97]
		Suppressed the activation of NF-κB, ↓ iNOS, TGF-β1, ALP, SGOT and SGPT.	HFD-STZ-induced type 2 diabetic Wistar rats	Ahad 2014 [99]
		Reduced AGEs and TNF-α level, decreased NF-κB activation.	STZ-induced diabetic rats	El-Bassossy 2014 [100]
		Improvement of insulin resistance, protective by phosphorylating AMPKα AND INS-1.	HFD-induced mice	Pu 2012 [101]
		Restored the impairment of PI3K/Akt pathway and ↓ GSK3β.	STZ-induced diabetic Wistar rats	Qi 2015 [102]
Chrysin	Honey, *Passiflora caerulea* (L.), *Pelargonium peltatum* (L.), *Tilia tomentosa* Moench, *Pelargonium quercifolium* (L.f.) L’Hér. and *Pelargonium crispum (Berg.)* L’Her	Inhibition of TNF-α pathway, leads to the decreased secretion of pro-inflammatory cytokines.	HFD-STZ-induced type 2 diabetic Wistar albino rats	Ahad 2014 [107]
		Downregulated the increased expression of TGF-β, fibronectin and collagen-IV proteins.		
		↓ Blood glucose, oxidative stress, improved learning and memory function.	STZ-induced diabetic rats	Li 2014 [108]
Luteolin	Celery, parsley, broccoli, onion leaves, carrots, peppers, cabbages and apple skins.	inhibition of the NF-κB pathway.	HFD-induced in obesity mice	Liu 2014 [116]
		Increased HO-1 expression and elevated antioxidants.	STZ-Induced Diabetic Rats.	Wang 2011 [117]
		Decreased activity of NF-κB was implicated in inhibition by luteolin of increased iNOS.	Min6 insulin secreting cell line	Ding 2014 [119]
		Reduced CREB-binding protein/p300 gene expression.	Human monocytic (THP-1) cell line	Kim 2014 [120]
		Suppression of hepatic lipogenesis and increased in uptake of FFAs.	HFD-induced C57BL/6 J mice	Kwon 2015 [121]
		Up-regulated the myocardial eNOS pathway and downstream effects include the enhancement of MnSOD and inhibition of mPTP.	STZ/L-NAME-induced diabetes rats	Yang 2015 [122]
		Reduced mast cell and macrophage infiltrations and inflammatory cytokine levels.	Diet-induced obesity	Xu 2014 [123]
Tangeretin	Citrus fruit rinds, mandarin orange and *Poncirus trifoliate* (L.) Raf.	Stimulated AMPK activation may be associated with anti-inflammatory.	HFD-induced obese mice	Kim 2012 [124]
		↑ Insulin, glycogen.	STZ-induced diabetic rats	Sundaram 2014 [125]
Wogonin	*Scutellaria baicalensis* Gerogi	Inhibition of p38 MAPK by its specific inhibitor SB203580 increasing PPARα activity and decreasing OPN expression.	STZ induced type 1 diabetes	Zhang 2015 [130]
		Anti-adipogenic effect by acting as a PPARα agonist, which could prevent weight gain.	C57BLKS/J-Leprdb/Leprdb mice and 3 T3-L1 cells	Bak 2014 [128]
Isorhamnetin	*Hippophae rhamnoides* L., *Oenanthe javanica* (Blume) DC, *Ginkgo biloba* L., *and Opuntia ficus-indica* (L.) Mill.	Insulin secretion, associated with increased GLUT2 and PPARγ.	HFD-induced C57BL/6 mice	Rodríguez-Rodríguez 2015 [133]
		Inhibition adipogenesis through downregulation of PPARγ and C/EBPα.	3 T3-L1 cells	Lee 2009 [134]
Kaempferol	Tea, cruciferous vegetables, grapefruit, *Gingko biloba* L., and some edible berrie.	Inhibited cellular apoptosis, and reduced caspase-3 activity in beta-cells.	INS-1E β-cells	Zhang 2011 [139]
		↑ Antioxidant and ↓ decreased of lipid peroxidation markers.	STZ-induced diabetic rats	Al-Numair 2015 [141]
		↓ PPAR-γ and SREBP-1c expression.	HFD-obese mice	Zang 2015 [143]
		Restore deranged activity of membrane-bound ATPases.	STZ-induced diabetes	Al-Numair 2015 [144]
		Enhancing β-cell survival, improved cAMP signaling.	INS-1E cells.	Zhang 2013 [145]
		↑ GLUT 4, AMPK	HFD-induced diabetic mice	Alkhalidy 2015 [146]
Rutin	Buckwheat, oranges, grapes, lemons, limes, peaches and berries	Inhibited inflammatory cytokines, improving antioxidant and lipid profiles.	HFD-STZ-induced type 2 diabetic model	Niture 2014 [154]
		↓ Glucose, TBARS, caspase-3 and ↑ insulin, Bcl-2 protein.	STZ-induced diabetic rat retina	Ola 2015 [156]
		Protected pancreatic beta-cell by decreasing oxidative stress.	STZ induced diabetic rats	Kamalakkannan and Prince 2006 [157]
		↓ MDA levels and ↑ SOD and CAT.	STZ-induced type 1 diabetic rats	Butchi 2011 [158]
Quercetin	Chokeberries, black currants, apples and cherries	Increased the activity of glycogen synthase, the rate-limiting enzyme of glycogen synthesis.	Murine H4IIE and human HepG2 cells.	Eid 2015 [164]
		Inhibition of the two transcriptional factors and the activation of mTORC1/p70S6K.	HK-2 and NRK-52E cells	Lu 2015 [167]
		Inhibitory effects on NF-kB and caspase-3 expression.	STZ-induced diabetic rats	Kumar 2014 [168]
		Ameliorated hyperglycemia and oxidative stress.	Alloxan induced type 2 diabetic mice.	Alam 2014 [169]
		Prevented β-cell death via the mitochondrial pathway and NF-κB signaling.	RINm5F β-cells.	Dai 2013 [170]
		Reduced expression of inducible iNOS and inhibited translocation of NF-κB.		
		Reduced TBARS levels, TC and elevated activities of SOD, CAT, and GSH-Px and HDL-cholesterol.	Diet-C57BL/KsJ-db/db mice	Jeong 2012 [171]
		Improved renal function in rats with diabetic nephropathy by inhibiting the overexpressions of TGF-β1 and CTGF.	STZ-induced diabetic rats	Lai 2012 [172]
		↓ Glucose and blood HbA1c.	STZ-induced diabetic rats	Kim 2011 [173]
Genistein	Fava bean, soybeans and kudzu.	↑ cAMP signalling ↑ PKA activation.	HG-induced diabetic mice	Babu 2012 [176]
		↑ Insulin-positive β-cell.	HFD-induced C57BL/6 mice	Fu 2012 [179]
		Activation of ERα seems to stimulate muscular GLUT4 functionality, activation of ERβ.	Zucker diabetic fatty rats	Weigt 2015 [182]
		↓ Glucose, HbA1c, C-reactive protein, TNFα and TGFβ1 protein expressions.	STZ-induced diabetes rats	Gupta 2015 [183]
		↓ Inflammatory markers and improved oxidative stress.	Alloxan-induced diabetic mice	Kim and Lim 2013 [184]
		Improved wound angiogenesis.	STZ-induced type 1 diabetic mice	Tie 2013 [185]
		Reduced hyperglycemia via minimization of islet cell loss.	Alloxan-induced Sprague–Dawley rats	Yang 2011 [186]
		Reduced glucose tolerance and improved insulin levels.	STZ-induced diabetic mice	Fu 2010 [187]
		Inhibition the secretion of ECM components and the expression of TGF-beta.	HG-cultured rat mesangial cells	Yuan 2012 [188]
		Suppressed the expression of CCAAT/enhancer binding protein alpha (C/EBPalpha).	3 T3-L1 cells	Zhang 2009 [189]
		↓ TGF-β2, αB-crystallin, and fibronectin.	Human lens epithelial (HLE-B3) cells	Kim 2008 [190]
		↓ G6Pase, PEPCK and ↑ lipogenic enzymes activities.	Non-obese diabetic mice	Choi 2008 [191]
Daidzein	Soy milk, soybeans and nuts	Potent α-glucosidase inhibitor and suppress the postprandial hyperglycemia.	STZ-induced diabetic mice	Park 2013 [194]
		↓ Blood glucose and urinary glucose excretion.	HFD-induced type 2 diabetes	Cheong 2014 [196]
		Improved the endothelial dysfunction.	STZ-induced diabetic rats.	Roghani 2013 [197]
		↑ IRS-1, GLUT4 and enhanced insulin stimulated glucose uptake.	3 T3-L1and C3H10T1/2 cells	Cho 2010 [198]

### Diosmin

Diosmin was first isolated in 1925 from *Scrophularia nodosa* L. Diosmin is a naturally occurring flavonoid glycoside that can be isolated from various plant sources or derived by dehydrogenation of the corresponding flavanone glycoside hesperidin that is abundant in the pericarp of various citrus fruits [28]. Diosmin has been shown to improve factors associated with diabetic complications. Blood parameters of glycation and oxidative stress was measured in type I diabetic patients before and after intervention with a diosmin. A decrease in glycated hemoglobin (HbA1c) was accompanied by an increase in glutathione peroxidase (GPx) [29]. In studies with rats, orally treatment of diosmin for 45 days significantly lowered plasma glucose level, increased the activities of hepatic key enzymes such as hexokinase and glucose-6- phosphate dehydrogenase (G6PD) in addition to decreasing glucose-6-phosphatase (G6Pase) and fructose-1,6-bisphosphatase (FDPase) in streptozotocin (STZ)-nicotinamide treated rats exhibiting its anti-hypeglycemic activities [30]. Studies from the same author claimed that, diosmin lowered plasma glucose and increased plasma insulin levels in diabetic rats by ameliorating the oxidative stress induced by STZ-nicotinamide and the activities of antioxidant enzymes such as superoxide dismutase (SOD), catalase (CAT), GPx, and reduced glutathione (GST), vitamin C, vitamin E and reduced glutathione were increased while lipid peroxidation was reduced in liver and kidney of diabetic rats upon treatment with diosmin [31]. In recent study, treatment with diosmin at doses of 50 and 100 mg/kg bw for 1 month ameliorated hyperglycemia and oxidative stress [32].

### Fisetin

Fisetin is a flavonoid, also a dietary ingredient found strawberry, apple, persimmon, grape, onion, cucumber and *Cotinus coggygria* Scop*.* [35, 36]. The results of Constantin et al. [37] showed that the fisetin inhibition of pyruvate transport into the mitochondria and the reduction of the cytosolic NADH-NAD ^(+)^ potential redox could be the causes of the gluconeogenesis inhibition. Fisetin could also prevent hyperglycemia by decreasing glycogen breakdown or blocking the glycogenolytic action of hormones. Fisetin was also reported that at a dose of 10 mg/kg bw to diabetic rats for 30 days, displayed reductions in blood glucose, HbA1c levels, increased plasma insulin and decreased mRNA and protein expression levels of gluconeogenicgenes, such as phosphoenol pyruvate carboxykinase and G6PD in liver [38]. There is evidence based *in vivo* animal studies showed that fisetin treatment significantly decreased the levels of blood glucose, HbA1c, NF-κB p65 unit and interleukin-1 beta (IL-1β), serum nitric oxide (NO) with an improved in plasma insulin antioxidant status [39]. Moreover, fisetin inhibits high glucose (HG)-induced cytokine production in monocytes, through epigenetic changes involving NF-κB. Thus, fisetin supplementation could be considered for diabetes prevention [40].

### Morin

Morin, a natural flavonoid, and a major component of traditional medicinal herbs were *Prunus dulcis* (Mill.) D.A. Webb*, Chlorophora tinctoria* (L.) Gaud*., Psidium guajava* L., fruits and wine [47, 48]. In animal models, oral administration of morin for 30 days significantly improved hyperglycemia, glucose intolerance, and insulin resistance. The elevated levels of lipid peroxides were declined and the antioxidant competence was found to be improved in diabetic rats treated with the morin. The status of the lipid and lipoprotein profile in the serum was normalized upon treatment. Levels of TNF*α* decreased upon treatment with morin [49]. A study carried out by Abuohashish et al., [50] demonstrated that morin (30 mg/kg bw) was effective in reducing the elevated inflammatory cytokines IL-1β, IL-6 and TNF-α in diabetic animals, which support its anti-inflammatory property and also its possible beneficial effects in diseases where inflammation. Morin was found to ameliorate high fructose-induced hepatic SphK1/S1P signaling pathway impairment, resulting in the reduction of hepatic NF-κB activation with IL-1b, IL-6 and TNF levels in rat liver and BRL3A cells. Subsequently, morin recovered hepatic insulin and leptin sensitivity, then reduced hyperlipidemia and liver lipid accumulation in animal and cell line models [51]. Paoli et al. [52] reported that dietary morin inhibitor of PTP1B, which behaves as an activator and sensitizer of the insulin receptor stimulating the metabolic pathways. However, in this context Vanitha et al. [53] showed that morin treatment significantly reduced the blood glucose, G6Pase and FDPase and increased the insulin levels, hexokinase and G6PD activities. Thus, morin, through these metabolic effects, may exert several beneficial effects in the prevention of diabetes.

### Eriodictyol

Eriodictyol is present in lemon fruit. It has been demonstrated [55] that supplementation of lemon flavonoids, such as eriocitrin and hesperidin, significantly suppressed the oxidative stress in diabetic rats. Eriodictyol treatment was associated with up-regulated the mRNA expression of PPAR*γ*2 and adipocyte-specific fatty acid-binding protein as well as the protein levels of PPAR*γ*2 in differentiated 3 T3-L1 adipocytes. Furthermore, it reactivated Akt in HepG2 cells with HG-induced insulin resistance [56]. Eriodictyol has been reported to significantly lower retinal TNF*α*, intercellular adhesion molecule 1 (ICAM-1), vascular endothelial growth factor (VEGF), and endothelial NOS (eNOS). Further, treatment with eriodictyol significantly suppressed diabetes-related lipid peroxidation [57].

### Hesperidin

Hesperidin is an abundant and inexpensive by product of Citrus cultivation and isolated from the ordinary orange *Citrus aurantium* L., and other species of the genus Citrus (family: Rutaceae) contain large amounts of hesperidin [58, 59]. Hesperidin not only attenuated the diabetic condition but also reversed neuropathic pain via control over hyperglycemia as well as hyperlipidemia to down-regulate generation of free radical, release of pro-inflammatory cytokines [60]. Reduced oxidative stress by hesperidin was also noted in this studies [61, 62]. Treatment of hesperidin (10 g/kg diet) decreased blood glucose by altering the activity of glucose regulating enzymes, and normalized the lipids and adiponectin levels [63]. In a recent study, Yo et al. [64] found that hesperidin treatment significantly improved mean arterial pressure, reduced left ventricular end-diastolic pressure, and improved both inotropic and lusitropic function of the heart. Furthermore, hesperidin treatment significantly decreased the level of thiobarbituric acid reactive substances (TBARS) and increased the activity of lactate dehydrogenase (LDH).

### Naringenin

Naringenin is abundantly found in citrus fruits such as grapefruits, oranges and tomatoes that has been reported to have antioxidant potential [70]. *In vitro* studies have shown that naringenin had an insulin-mimic effect to decrease poliprotein B secretion in hepatocytes [71]. *In vivo* studies of *Cochlospermum vitifolium* (Willd.) Spreng which contains naringenin decreased blood glucose levels in healthy male Wistar rats [72]. Oral treatment of naringenin (25 mg/kg bw) exerts significant inhibition of intestinal α-glucosidase activity *in vivo* thereby delaying the absorption of carbohydrates in diabetic rats, thus resulting in significant lowering of postprandial blood glucose levels. Both *in vitro* and *in vivo* results were compared to the commercially available α-glucosidase inhibitor acarbose [73]. Naringenin was found to inhibit the glucose uptake in everted rat intestinal sleeves by the inhibition of intestinal sodium-glucose co-transporters [74]. Naringenin was also found to prevent the functional changes in vascular reactivity in diabetic rats through nitric oxide and not prostagland in dependent pathway [75]. Naringin is effective in protecting against the development of metabolic syndrome through changing the expression of hepatic genes involved in lipid metabolism and gluconeogenesis via upregulation of both PPAR and 5' adenosine monophosphate-activated protein kinase (AMPK), involving the activation of multiple types of intracellular signaling in mice exposed to a HFD [76]. The research findings of Zygmunt et al. [77] provide support for the stimulation of muscle glucose uptake by naringenin in a dose-dependent manner and independent of insulin. The in vivo anti-diabetic effects of naringenin may be AMPK-mediated. Activation of AMPK increases glucose tolerance, insulin sensitivity. Furthermore, naringenin administration decreased plasma glucose levels in STZ-induced diabetic rats [78], improved insulin sensitivity in fructose-fed insulin resistant rats [79], and reduced insulin resistance in HFD mice [80]. A recent study stated that naringenin (25 mg/kg bw) treatment to diabetic rats for 45 days markedly reduced hyperglycemia and hyperinsulinemia, restored lipid profile changes, decreased membrane lipid peroxidation; enhanced the activities of antioxidants and improved hepatic function markers [81].

### Apigenin

Apigenin is a member of the flavone family and is found in many fruits, vegetables, nuts, onion, orange and tea [87]. Alloxan-induced elevation in serum cholesterol, hepatic lipid peroxidation and a decrease in the activity of cellular antioxidants, such as CAT, SOD and GSH content were observed, administration of apigenin to diabetic mice ameliorated hyperglycemic and improved antioxidants [88]. Apigenin reduced parathyroid hormone related protein stimulated increases in the human pancreatic stellate cells messenger RNA expression levels of extracellular matrix proteins collagen 1A1 and fibronectin, proliferating cell nuclear antigen, TGF-β, and IL-6 [89]. Suh et al. [90] suggest that apigenin attenuates dRib induced cell damage in pancreatic *β*-cells via oxidative stress-related signaling. Apigenin preserves the cellular architecture of vital tissues towards normal in STZ-induced diabetic rats. Furthermore, enhanced GLUT4 translocation upon apigenin treatment suggests more glucose lowering as well as β-cell preserving efficacy [91].

### Baicalein

Baicalein, a flavonoid originally isolated from the roots of *Scutellaria baicalensis* Georgi and fruits of *Oroxylum indicum* (L.) Benth has been shown to exhibit strong free radical scavenging [95, 96]. Fu et al. [97] induced diabetes mice by HFD feeding and low doses of STZ and they the administered HF diet containing 0.25 or 0.5 g baicalein/kg diet. They observed that diabetic mice treated with baicalein displayed significantly improved hyperglycemia, glucose tolerance, and insulin levels. They provide the rationale for screening and preclinical studies of hydroxyflavones, especially those with an improved pharmacological profile, as potential therapeutics for diabetic ant its complications [98]. Baicalein treatment significantly lowered food intake, body weight and levels of fasting blood glucose, HbA1c in diabetic rats. Baicalein also suppressed the activation of NF-κB, decreased expression of iNOS and TGF-β1, and ameliorated the structural changes in renal tissues [99]. Previous findings also reported that treatment with baicalein reduced the advanced glycation end-product (AGEs) and TNF level, decreased NF-κB activation and inhibited histopathological changes [100]. Mechanism of its action was up-regulation of AMPK and its related signal pathway. AMPK is not only a major cellular energy sensor, but also a master regulator of metabolic homeostasis involving inflammation and oxidative stress. Activated AMPK could abolish inflammation through the MAPKs signaling pathway; activated AMPK could attenuate insulin resistance by phosphorylating IRS-1, AKT and dephosphorylate ERK, JNK and NF-κB; it also suppresses fatty acid synthesis, gluconeogenesis and increases mitochondrial *β*-oxidation [101].

### Chrysin

Chrysin, found in honey, bee pollen, propolis, fruits, vegetables, beverages and medicinal plants such as *Passiflora caerulea* (L.), *Pelargonium peltatum* (L.), *Tilia tomentosa* Moench, *Pelargonium quercifolium* (L.f.) L’Hér and *Pelargonium crispum (Berg.)* L’Her [103–106]. Chrysin is the main component of *Oroxylum indicum* (L.) Benth. ex Kurz, which is one of the most common herbal medicines used in China and other East Asian countries. Ahad et al. [107] in a recent study mentioned that chrysin treatment improved renal pathology and suppressed TGF-β, fibronectin and collagen-IV protein expressions in renal tissues. Chrysin also significantly reduced the serum levels of pro-inflammatory cytokines, IL-1β and IL-6. Therefore, chrysin prevents the development of diabetic neuropathy (DN) in HFD/STZ-induced diabetic rats through anti-inflammatory effects in the kidney by specifically targeting the TNF-α pathway. Li et al., [108] investigation revealed that chrysin significantly and dose-dependently inhibited the oxidative stress, together with improved cognition in diabetic rats. In addition, treatment chrysin was shown to reduce glucose and lipid peroxidation level and improve insulin levels in diabetic rats [109], suggesting that this chrysin may exert anti-hypertensive and vascular complication associated with anti-diabetic effects [110].

### Luteolin

Vegetables and fruits such as celery, parsley, broccoli, onion leaves, carrots, peppers, cabbages, apple skins, and chrysanthemum flowers are rich in luteolin [111–114]. That luteolin potentiates insulin action, increases expression and transcriptional activation of PPARγ and expression of the PPARγ target genes adiponectin, leptin and GLUT4 in 3T3-L1 adipocytes, as well as in primary mouse adipose cells, and that PPARγ antagonist inhibits this induction [115]. It has also been reported that luteolin up-regulated the expression of the synaptic proteins, and alleviated the HFD-induced cognitive deficits. It is possible that the decrease in circulating levels of inflammatory molecules MCP-1, resistin and the elevation of adiponectin levels in obese mice by luteolin may, in turn, mediate beneficial effects on metabolic pathways implicated in insulin resistance and DM pathophysiology [116]. The mechanism of the renoprotective effect of luteolin may be related to increasing HO-1 expression and elevating antioxidant in diabetic nephropathy [117]. It has been reported that luteolin ameliorated inflammation related endothelial insulin resistance in an IKKb/IRS-1/Akt/eNOS-dependent pathway [118]. Luteolin, also improved insulin secretion in uric acid damaged pancreatic β-cells by suppressing the decrease of MafA mainly through the NF-κB, iNOS-NO signaling pathway [119]. In addition, it has been reported that luteolin was significantly reduced cAMP-response element binding protein (CREB)-binding protein/p300 gene expression, as well as the levels of acetylation and histone acetyl transferase activity of the CBP/p300 protein, which is a known NF-κB coactivator [120].

### Tangeretin

Tangeretin, abundant in the citrus fruit rinds, including mandarin orange, *Poncirus trifoliate* Raf. (Rutaceae), and *Yuja*, found in Korea. Administration of HFD plus 200 mg/kg bw of tangeretin exhibited reduction in body weight, total cholesterol (TG), blood glucose and decreased adipocytokinese such as adiponectin, leptin, resistin, IL-6, and MCP-1 [124]. Oral administration of tangeretin (100 mg/kg bw) to diabetic rats for 30 days resulted in a significant reduction in the levels of plasma glucose, HbA1c and increased in the levels of insulin and hemoglobin. Tangeretin enhances the glycolytic enzymes and controls the glucose metabolism in the hepatic tissues of diabetic rats by stimulating insulin production from existing *β*-cells of pancreas by its antioxidant potential [125]. Tangeretin 3T3-L1 preadipocytes into adipocytes possessing less intracellular triglyceride as compared to vehicle-treated differentiated 3T3-L1 adipocytes. Tangeretin increased the secretion of an insulin-sensitizing factor, adiponectin, but concomitantly decreased the secretion of an insulin-resistance factor, monocyte chemotactic protein-1 (MCP-1), in 3T3-L1 adipocytes [126].

### Wogonin

Wogonin extracted from the root of *Scutellaria baicalensis* Gerogi (*Scutellariae* radix) has long been used as a traditional medicine in East Asian countries [127]. Wogonin has beneficial effects on blood glucose level, insulin sensitivity, and lipid metabolism via selective PPARα and AMPK activation without the adverse side-effects of weight gain and fatty liver [128]. High glucose (HG)-induced markedly increased vascular permeability, monocyte adhesion, expressions of cell adhesion molecules, formation of ROS and activation of NF-κB. Remarkably, all of the above mentioned vascular inflammatory effects of HG were attenuated by pretreatment with wogonin [129].

### Isorhamnetin

Isorhamnetin is a bioactive compound found in medicinal plants, such as *Hippophae rhamnoides* L., *Oenanthe javanica* (Blume) DC., and *Ginkgo biloba* L. In a STZ-induced model of diabetes, oral administration of isorhamnetin (10, 20 mg/kg BW) for 10 days ameliorated hyperglycemia and oxidative stress [131]. In another study, administered orally at 25 mg/kg in STZ-induced diabetic rats caused not only a significant inhibition of serum glucose concentration but also sorbitol accumulation in the lenses, red blood cells, and sciatic nerves [132]. In recent year, there is experimental evidence suggesting that isorhamnetin glycosides may possess the antidiabetic effect and their influence on lipid content, endoplasmic reticulum stress markers and the expression of enzymes regulating lipid metabolism [133].

### Kaempferol

Kaempferol is a flavonol that is relatively abundant in *Ginkgo biloba* L., cruciferous vegetables, grapefruit, tea and some edible berries [135–137]. Also, kaempferol is isolated from *Bauhinia forficata* leaves, is able to diminish the increased serum glucose level and increase glucose uptake in the rat soleus muscle as efficiently as insulin [138]. The in vitro results demonstrated that kaempferol treatment (10 μM) promoted viability, inhibited cellular apoptosis, and reduced caspase-3 activity in *β*-cells and human islets chronically exposed to hyperglycemic condition. These protective effects are associated with improved cAMP signaling, anti-apoptotic Akt and Bcl-2 protein expression, and insulin secretion and synthesis in *β*-cells [139]. The author suggests that kaempferol stimulates glucose uptake in the rat soleus muscle via the PI3K and PKC pathways and, at least in part, independently of MEK pathways and the synthesis of new glucose transporters [140]. Administration of kaempferol to diabetic rats was shown back to near normal levels in plasma glucose, insulin, lipid peroxidation products, enzymatic, and non-enzymatic antioxidants [141]. Published data showed that kaempferol reduced IL-1β, TNF-α, lipid peroxidation and nitrite, concomitant with the improvement of antioxidant defense and body weight gain [142]. Orally administrated kaempferol was significantly decreased fasting blood glucose, serum HbA1c levels and improved insulin resistance. Gene expression analysis of the liver showed that kaempferol decreased PPAR-γ and SREBP-1c expression. Anti-obese and anti-diabetic effects of kaempferol are mediated by SREBP-1c and PPAR-γ regulation through AMPK activation [143].

### Rutin

Rutin can be broadly extracted from natural sources such as buckwheat, oranges, grapes, lemons, limes, peaches and berries [147, 148]. Specifically, diabetic mice fed rutin at 100 mg/kg diet displayed significant lower of plasma glucose and increase in insulin levels were observed along with the restoration of glycogen content and the activities of carbohydrate metabolic enzymes [149]. Fernandes et al. [150] showed that rutin could improve the metabolic status of rats with experimentally-induced diabetes. Rutin is involved in the activation of liver enzymes associated with gluconeogenic, and lipid metabolic processes [151] and also decreased the levels of fasting blood glucose, creatinine, blood urea nitrogen, urine protein, the intensity of oxidative stress and p-Smad 7 significantly. The expression of AGEs, collagen IV and laminin, TGF-β1, p-Smad 2/3 and connective tissue growth factor was inhibited by rutin significantly [152]. Rutin have been shown to stimulate glucose uptake in the rat soleus muscle via the PI3K, a typical protein kinase C and mitogen-activated protein kinase pathways [153].

Rutin has been reported to significantly improved body weight, reduced plasma glucose and HbA1c, pro-inflammatory cytokines (IL-6 and TNF-alpha), and restored the depleted liver antioxidant status and serum lipid profile in HFD/STZ induced diabetic rats [154]. Notably, rutin was shown to protect and improve myocardial dysfunction, oxidative stress, apoptosis and inflammation in the hearts of the diabetic rats [155].

A recent study showed that rutin supplementation enhanced the reduced levels of brain-derived neurotrophic factor, nerve growth factor, and GSH, and reduced the level of TBARS. In addition, rutin treatment showed anti-apoptotic activity by decreasing the level of caspase-3 and increasing the level of Bcl-2 in the diabetic retina [156].

### Quercetin

Numerous studies have focused on quercetin to develop it as antidiabetic drug to prevent and manage DM. Quercetin is one of the most widely used flavonols in human dietary nutrition [159]. It is widely distributed in different types of fruits, tea, lovage, pepper, coriander, fennel, radish, dill, berries, onions, apples and wine [160]. Several studies have reported quercetin mechanism of action in diabetes, such as decreases in lipid peroxidation, increases in antioxidant enzymes (like SOD, GPX, and CAT) activities, inhibition of insulin-dependent activation of PI3K, and reduction in intestinal glucose absorption by inhibiting GLUT2 [161, 162]. The study performed by Kwon et al. [163] evaluated the effect of quercetin on Caco-2E intestinal cells, the study documented that the transport of fructose and glucose by GLUT2 was strongly inhibited by quercetin. Blockage of tyrosine kinase is another mechanism by which quercetin is reported to have effects against diabetes. Eid et al. [164] reported that quercetin stimulates GLUT4 translocation and expression in skeletal muscle, by mechanisms associated with the activation of AMPK rather than insulin-dependent pathways such as Akt. The study performed in rats indicated that quercetin ameliorated the expression of markers of oxidative stress and inflammation, such as Nrf2, heme oxygenase-1, and NF-κB, suggesting that quercetin anti-inflammatory effects on adipose tissue can be associated with the reduction of body weight [165]. Likewise, Kobori et al. [166] reported that quercetin in the diet led to the recovery of cell proliferation in diabetic mice.

### Isoflavones

Isoflavonoids are another subclass of the phenolic phytonutrients. Soybeans are an unusually concentrated source of isoflavones, including genistein and daidzein, and soy is the major source of dietary isoflavones, is reported to have numerous health benefits attributed to multiple biological functions. Over the past 10 years, numerous studies have demonstrated that isolavones has anti-diabetic effects, in particular, direct effects on β-cell proliferation, glucose-stimulated insulin secretion.

### Genistein

Genistein, a naturally occurring soy isoflavone, is a flavonoid presented in legumes and Chinese plants *Genista tinctoria* Linn and *Sophora subprostrala Chun* et T Chen [174]. Molecular mechanisms underlying the modulatory effect of genistein on diabetes, that specifically focus on neutrophils, are needed to understand contributions of estrogenic and enzyme inhibitory activities (tyrosine kinase inhibition) to dysregulat glucose homeostasis [175]. Genistein has also been reported to improve hyperglycemia caused human vascular endothelial inflammation ex *vivo*, which is at least partially mediated through promoting the cAMP/PKA signaling pathway [176]. Palanisamy et al. [177], focused on the protective role of genistein on renal malfunction in rats fed a fructose rich diet, through the modulation of insulin resistance induced pathological pathways. Another study has shown that genistein injections (10 mg/kg) reduced urinary TBARs excretion and renal gp91phox expression, as well as decreased production of inflammatory markers, including p-ERK, ICAM-1, and MCP-1, in DN mice [178]. In this study, dietary intake of genistein (250 mg/kg bw) improved hyperglycemia, glucose tolerance, and blood insulin level in obese diabetic mice, whereas it did not affect body weight gain, food intake, fat deposit, plasma lipid profile, and peripheral insulin sensitivity [179]. Furthermore, genistein has been shown to protect against oxidative stress and inflammation, neuropathic pain, and neurotrophic and vasculature deficits in the diabetic mouse model [180]. Indeed, recent findings indicated that genistein administration significantly decreased *β*-cells loss and improved glucose and insulin levels [181].

Daidzein belongs to the isoflavone subclass of flavonoids and is found in fruits, nuts, soybeans, and soy-based products [192]. An earlier study suggested that daidzein exerts anti-diabetic effects by improves glucose and lipid metabolism [193, 194]. Cederroth et al. [195] demonstrated that the dietary soy including genistein improve insulin sensitivity by increasing glucose uptake in skeletal muscle in mice. Treatment of daidzein proved to be effective in reducing blood glucose; TC levels and improved the AMPK phosphorylation in gastrocnemius muscle [196].

### Anthocyanins

Anthocyanins are flavonoids in flowers and fruits (red, blue and purple tints in apples, berries, red grapes, eggplant, red cabbage and radishes). Dietary consumption of anthocyanins is high compared to other flavonoids, owing to their wide distribution in plant materials. Based upon many cell-line studies and animal models, it has been suggested that anthocyanins possess anti-diabetic activities (Table 2).

**Table 2:** The *in vitro* and *in vivo* effect on anti-diabetic and underlying mechanism of anthocyanins

Strcture of anthocyanins	Plants/dietary source	Specific mechanism of action	Model	References
Cyanidin	Grapes, bilberry, blackberry, blueberry, cherry, cranberry, elderberry, hawthorn, logan berry, acai berry and raspberry.	↑ pAMPK, pACC signaling and improve insulin signaling (pAkt, pFOXO-1).	HFD-induced obesity rats	Park 2015 [206]
		↑ PGC-1α, SIRT1 and UCP-3 genes.	3 T3-Ll cells	Matsukawa 2015 [207]
		Lowered fasting glucose and improved insulin sensitivity.	C57BL/6 J obese mice	Guo 2012 [205]
		Decreased c-Jun N-terminal kinase activation and FoXO1.		
		Upregulated the GLUT4 and down-regulation of the inflammatory adipocytokines.	HFD-KK-A(y) mice	Sasaki 2007 [208]
		Suppressed the mRNA levels of enzymes involved in FA and TG synthesis and lowered the SREBP-1 level.	High fat-induced diabetic mice	Tsuda 2003 [209]
		↓ Glucose, mitochondrial (ROS)	INS-1 cells and STZ-induced diabetic mice	Sun 2012 [210]
Delphinidin	Berries, dark grapes and vegetables such as eggplant, tomato, carrot, purple sweet potato, red cabbage and red onion	↓ Albumin and HbA1c glycation.	Diabetic rats	Gharib 2013 [212]
		Cyclooxygenase inhibitor restored the relaxant responses to Ach and SNP.	Diabetic microangiopathy.	Bertuglia 1995 [211]
Pelargonidin	*Ficus bengalensis* Linn and billberry	↓ Glucose, TBARS and ↑ SOD	STZ-injected diabetic rats	Mirshekar 2010 [215]
		Improved retention and recall capability.	STZ-diabetic rats	Mirshekar 2011 [217]

### Cyanidin

Cyanidin and its glycosides belong to anthocyanins, and are widely distributed in various human diets through crops, vegetables, fruits, and red wine suggesting that we daily ingest significant amounts of these compounds from plant-based diets. Cyanidin has been demonstrated to inhibit intestinal α-glucosidase and pancreatic α-amylase, which is one of the therapeutic approaches for treatment of DM [199]. Anthocyanin was also found to reverse degenerative changes in β-cells in STZ-induced diabetic rats by activating insulin receptor phosphorylation and preventing pancreatic apoptosis [200]. Nasri et al. [201] performed in vivo chronic treatment of diabetic rats with cyanidin-3-glucoside (C3G) could prevent the functional changes in vascular reactivity observed in diabetic rats through endothelium dependent pathways and via attenuation of aortic lipid peroxidation. C3G, one of the most prevalent anthocyanins existing in our diet, can protect hepatocytes against HG-induced damage by improving antioxidant status and inhibiting the mitochondria-mediated apoptotic pathway through activation of Akt and inactivation of JNK [202, 203] and another author reported that anthocyanins exert a hepatoprotective effect against hyperglycemia-accelerated steatohepatitis in NAFLD [204]. Moreover Guo et al. [205] showed that C3G significantly reduced macrophage infiltration and the mRNA levels of MCP-1, TNF-α and IL-6 in adipose tissue and phosphorylation of FoxO1 via the Akt-dependent pathway, and the extent of phosphorylation represents the FoxO1 transcriptional activity in liver and adipose tissues of HFD and db/db mice.

### Delphinidin

Delphinidin present in pigmented fruits such as pomegranate, berries, dark grapes and vegetables such as eggplant, tomato, carrot, purple sweet potato, red cabbage and red onion and it possesses strong antioxidant activities. Delphinidin was observed in vivo at the microcirculatory level prevent the injury to endothelial cell function associated with diabetes and/or oxidative stress [211]. Moreover, administration of 100 mg/kg delphinidin chloride-loaded liposomes to diabetic mice at 8 weeks could decrease the rate of albumin and HbA1c glycation [212].

### Pelargonidin

Pelargonidin can be found in berries such as ripe raspberries, blueberries, blackberries, cranberries and saskatoon berries [213] Pelargonidin treatment counteracts hyperglycemia and relieves the oxidative stress including hemoglobin (Hb) induced iron mediated oxidative reactions by lowering the glycation level and free iron of Hb [214]. Pelargonidin was also demonstrated to reduce TBARS formation and non-significantly reversed elevation of nitrite level and reduction of antioxidant defensive enzyme superoxide dismutase in diabetic rats [215]. Pelargonidin-3-galactoside and its aglycone stimulate insulin secretion in rodent pancreatic *β*-cells in vitro in presence of glucose [216].

## Conclusions

**Fig. 1:**
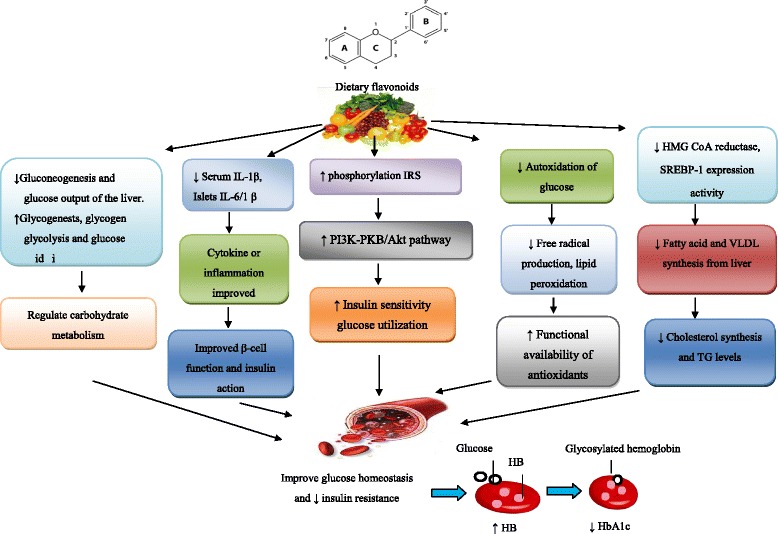
Schematic of the proposed role of flavonoids on management of blood glucose in diabetes. AKT; v-akt murine thymoma viral oncogene homolog, IRS; Insulin receptor substrate, HB; hemoglobin, HbA1c; Glycated hemoglobin, HMG-CoA :3-hydroxy-3-methylglutaryl-coenzyme A, IL-1β; Interleukin-1 beta, PI3K; Phosphatidylinositol-3-kinase, SREBP-1c; Sterol regulatory element-binding protein, TG; Triglycerides, VLDL; Very low density lipoprotein, (↑ Increase, ↓ Decrease)

The actual antidiabetic prospective associated with flavonoids are usually large as a result of their modulatory effects on blood sugar transporter by enhancing insulin secretion, reducing apoptosis and promoting proliferation of pancreatic β-cells, reducing insulin resistance, inflammation and oxidative stress in muscle and promoting translocation of GLUT4 via PI3K/AKT and AMPK pathways (Fig. 1). The molecular mechanisms underlying the glucose and lipid metabolism in diabetes would provide new insights in the field of drug development, continue to fuel excitement in this area of research and buoys the hope that future discoveries may one day yield therapeutic benefits. With the rapidly increasing incidence of diabetes worldwide, there is a greater need for safe and effective functional biomaterials with antidiabetic activity. Hence, meticulously intended human studies are needed to further measure the likely connected with a number of nutritional flavonoids to treat diabetes and its complication.

## Abbreviations

AGEs: advanced glycation end products
AKT: v-akt murine thymoma viral oncogene homolog
AMPK: 5′AMP-activated protein kinase
CAT: catalase
DM: diabetes mellitus
eNOS: endothelial nitric oxide synthase
FFA: free fatty acid
TG: triglycerides
GPx: glutathione peroxidase
GR: glutathione reductase
GST: glutathione S-transferase
HB: hemoglobin
HbA1c: glycated hemoglobin
HDL: high density lipoprotein
HFD: high-fat diet
HG: high glucose
HMG-CoA: 3-hydroxy-3-methylglutaryl-coenzyme A
ICAM-1: intercellular adhesion molecule 1
IL-1β: interleukin-1 beta
iNOS: inducible nitric oxide synthase
IR: insulin resistance
IRS-1: insulin receptor substrate-1
IRS-2: insulin receptor substrate-2
LDL: low density lipoprotein
MAPK: mitogen activated protein kinase
NF-κB: nuclear factor kappa B
NO: nitric oxide
PI3K: phosphatidylinositol-3-kinase
PPARγ: peroxisome proliferator-activated receptor gamma
ROS: reactive oxygen species
SOD: superoxide dismutase
SREBP-1c: sterol regulatory element-binding protein
STZ: streptozotocin
TNFα: tumor necrosis factor α
VEGF: vascular endothelial growth factor
VLDL: very low density lipoprotein
